# Investigation of Impact-Ionization-Enhanced Effect on SiC Thyristors Triggered by Weak UV Light

**DOI:** 10.3390/mi16070761

**Published:** 2025-06-29

**Authors:** Yulei Zhang, Xi Wang, Lechen Liu, Xuan Ji, Junhui Hou, Hongbin Pu

**Affiliations:** 1 Department of Electrical Engineering, Xi’an University of Technology, Xi’an 710048, China; zhangyulei_zl@163.com (Y.Z.); puhongbin@xaut.edu.cn (H.P.); 2 Xi’an Key Laboratory of Power Electronic Devices and High Efficiency Power Conversion, Xi’an 710048, China

**Keywords:** 4H-SiC, thyristor, light-triggered switches, UV light, impact ionization

## Abstract

The impact-ionization-enhanced mechanism is introduced into a SiC light-triggered thyristor (LTT) to improve its switching speed under weak UV illumination. The effects of impact ionization on photogenerated carrier multiplication and the dynamic switching performance of the SiC LTT are investigated through TCAD simulation. The relationships between bias voltage, UV light intensity, and key dynamic parameters are analyzed. Simulation results indicate that when the bias voltage exceeds 14 kV, the device enters the avalanche multiplication regime, leading to a significant increase in photocurrent under a given UV intensity. As the bias voltage increases, the turn-on time of the thyristor first decreases, then saturates, and finally drops rapidly. Under UV illumination of 100 mW/cm^2^, the turn-on time decreases from 10.1 μs at 1 kV to 0.85 μs at 18 kV, while the switching energy dissipation at 18 kV is only 1292.3 mJ/cm^2^. These results demonstrate that the impact-ionization-enhanced effect substantially improves the switching performance of SiC LTTs.

## 1. Introduction

As a result of the advantages of light-triggering mechanisms, such as simplified circuit design and enhanced electromagnetic interference (EMI) immunity, optically controlled switches are promising candidates for high-power applications [1,2]. Optically controlled solid-state switches feature a compact size and high repetition frequency, including photoconductive switches, optically triggered PIN diodes, optically activated transistors, and light-triggered thyristors (LTTs) [3,4,5,6]. Among these, LTTs offer high power density and low on-state resistance and have demonstrated significant progress [6,7].

As a typical wide-bandgap semiconductor material, silicon carbide (SiC) exhibits several advantages, such as a low intrinsic carrier concentration, high thermal conductivity, excellent temperature tolerance, and high critical avalanche breakdown electric field strength [4,5]. With the rapid development of SiC materials and device fabrication technologies in recent years, SiC LTTs with multilayer thick epitaxial structures operating at 10–18 kV have been demonstrated, laying the foundation for the development of ultrahigh-voltage SiC LTTs [8,9,10,11]. SiC shows great potential for ultra-high-voltage LTT fabrication, enabling a higher blocking voltage level, larger conduction current density, and faster switching speed [8,9,10]. If SiC-based LTTs can be used to replace Si-based LTTs, the number of series-connected switches can be significantly reduced, thereby improving the overall power density and system efficiency.

To switch on a SiC LTT, the positive feedback between the internally coupled npn and pnp transistors is essential [11,12]. Due to the low injection efficiency of the anode emitter and the large thickness of the long blocking base layer, both coupled transistors exhibit low current gain, which directly slows down the turn-on process of the SiC LTT [10,13]. As a result, high-power UV lasers are commonly used to trigger SiC LTTs in order to generate a higher optical current, thereby accelerating the positive feedback process and enhancing the di/dt capability in practical applications [14,15]. However, UV laser sources are typically bulky and inefficient, which limits the benefits of SiC LTTs in terms of system volume reduction and efficiency improvement. In contrast, UV light-emitting diodes (LEDs) offer advantages such as a small size, low cost, and ease of integration. Nevertheless, the relatively weak intensity of UV LEDs makes it challenging to rapidly trigger SiC LTTs under normal conditions [9,16]. To address this issue, many studies have been conducted, and the switching performance of SiC LTTs triggered by weak UV light has been significantly improved [11,13,17,18]. However, under weak illumination, the generated photocurrent remains inherently low, resulting in a turn-on time that is still far from ideal. Therefore, it is of great significance to explore effective methods for achieving fast switching of SiC LTTs under low-intensity optical triggering conditions.

In this work, the effects of photogenerated carrier multiplication caused by impact ionization on the fast switching behavior of SiC LTTs are investigated. The relationships between bias voltage, UV light intensity, and switching performance of SiC thyristors are systematically simulated and discussed.

## 2. Device Structure and Mechanism

**Figure 1:**
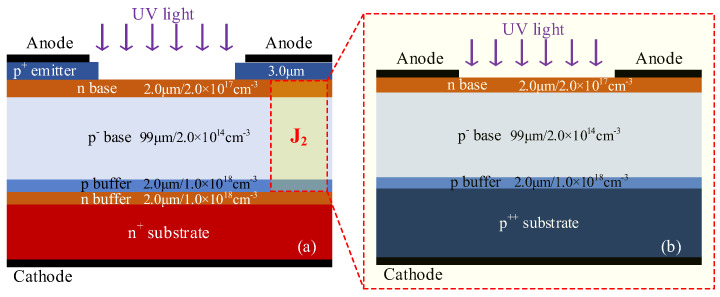
Structure of the device. (**a**) Cross-section structure of the SiC LTT. (**b**) Cross-section structure diagram of the inner diode formed by the J_2_ junction.

The cross-sectional structure of the 4H-SiC LTT is shown in Figure 1a. The thyristor was constructed with 6 layers as p^+^np^−^pnn^+^ in the vertical direction. The top p^+^ layer serves as the anode emitter region, with a thickness and doping concentration of 3.0 μm and 2.0 × 10^19^ cm^−3^, respectively. The p^+^ emitter is divided by optical windows into multiple bars that are uniformly distributed over the thin n-base. The widths of the p^+^ emitter and optical window are 12 μm and 8.0 μm, respectively. The subsurface n layer functions as a thin n-base region, with a thickness and doping concentration of 2.0 μm and 2.0 × 10^17^ cm^−3^, respectively. The p^−^ layer beneath it, which serves as the blocking base, has a thickness of 99 μm and a doping concentration of 2.0 × 10^14^ cm^−3^. The bottom p and n^+^ layers together form the cathode emitter junction. The thickness and doping concentration of the p buffer layer in the cathode are 2.0 μm and 1.0 × 10^18^ cm^−3^, respectively, and the n-type buffer layer shares the same parameters. The thickness and doping concentration of the n^+^ substrate are set to 150 μm and 2.0 × 10^18^ cm^−3^, respectively.

**Figure 2:**
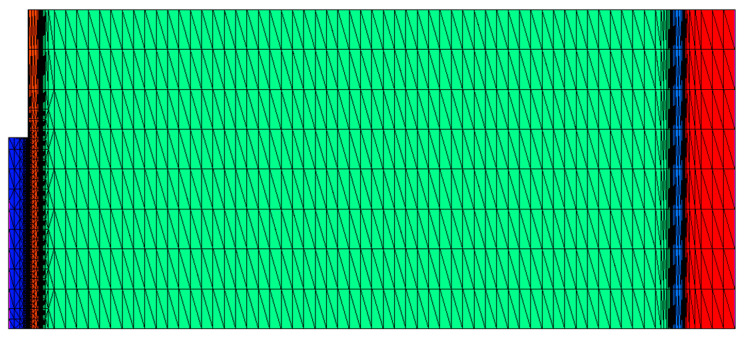
Mesh structure of the device unit cell.

When in the blocking state, the voltage is mainly applied across the J_2_ junction, which is composed of a thin n^−^ base and a thick p^−^ base. Since the doping concentration of the thick p^−^ base is much lower than that of the thin n^−^ base, the depletion region predominantly extends into the p^−^ base. Avalanche multiplication due to impact ionization primarily occurs within the p^−^ base. To analyze this mechanism, the thin n^−^ base and thick p^−^ base are extracted to construct a photodiode, as illustrated in Figure 1b. Except for the n^+^ buffer and n^+^ substrate, the structural parameters of the photodiode are identical to those of the J2 junction in the LTT. The main simulation models include the Caughey–Thomas model, the Canali mobility model [19], the incomplete ionization model [20], SRH recombination and Auger recombination models [21], the Okuto–Crowell collision ionization model [22], and the complex refractive index model [23]. Newton iteration combined with 128-bit extended-precision floating-point arithmetic was employed to solve the strongly coupled nonlinear semiconductor equations in this simulation. The maximum number of iterations per step was set to 45, with up to 50 undamped iterations allowed to enhance convergence under strongly nonlinear conditions such as avalanche breakdown. The effective error tolerances for electrons and holes were both set to 10^−32^ to ensure high numerical accuracy. To avoid numerical instability in low-carrier-density regions, a minimum carrier concentration threshold of 10^−30^ cm^−3^ was enforced. The mesh structure near the J_2_ junction is shown in Figure 2.

**Figure 3:**
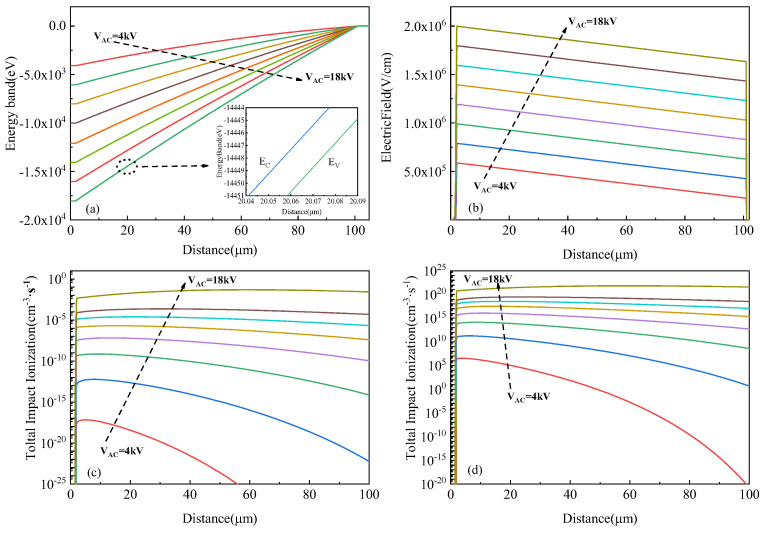
Energy band, electric field, and impact ionization rate distribution in the J_2_ junction at different blocking voltages. (**a**) Energy band. (**b**) Electric field. (**c**) Total impact ionization rate under dark conditions. (**d**) Total impact ionization rate under light conditions.

The electric field distribution and impact ionization rate under different reverse bias conditions were simulated using Sentaurus TCAD 2018, as shown in Figure 3. Partial code listings are available in Appendix A. A strong electric field is primarily concentrated in the space charge region near the J_2_ junction, which is also the main site where carrier impact ionization occurs, as illustrated in Figure 3a,b. Figure 3c presents the distribution of the total impact ionization rate under dark conditions. It is evident that the ionization rate increases significantly with the electric field intensity in the high-field region. As the reverse bias increases from 4 kV to 18 kV, the peak ionization rate under dark conditions increases by nearly ten orders of magnitude. Furthermore, Figure 3d shows the impact of ionization distribution under illumination. The introduction of photogenerated carriers enhances the local carrier density, and when combined with the strong electric field, it results in an exponential increase in the ionization rate within the ionization region. This synergistic mechanism significantly reinforces the avalanche multiplication process.

**Figure 4:**
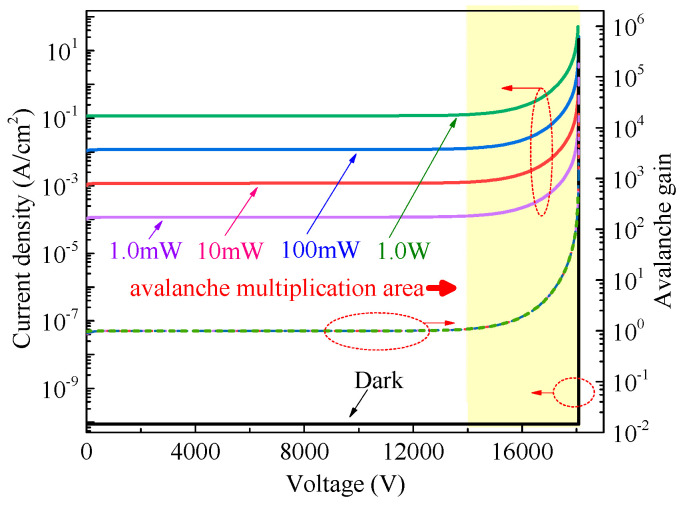
Avalanche gain characteristics of a photodiode formed by the J_2_ junction at various bias voltages.

The static current–illumination characteristic curves of the photodiode were simulated and are shown in Figure 4. The wavelength of the UV light used in this work is 365 nm. As shown in the figure, the blocking voltage of the photodiode exceeds 18 kV, and the current density remains low before breakdown under dark conditions. Under UV irradiation, the current density increases with increasing UV light intensity. At a specific UV light intensity, the photocurrent remains approximately constant when the bias voltage is lower than 14 kV. In this voltage region, as the incident UV power intensity increases from 1 mW/cm^2^ to 1 W/cm^2^, the photocurrent density exhibits a positive correlation with the light intensity. When the bias voltage exceeds 14 kV, the device enters the avalanche multiplication regime. In this regime, the photocurrent increases significantly with voltage under a fixed UV light intensity, mainly due to impact ionization of photogenerated carriers. To analyze the mechanism of photogenerated carrier multiplication, the avalanche gain of the photodiode can be expressed as follows [24]:

(1) $$M=\frac{I_{PM}-I_{DM}}{I_{P}-I_{D}}$$

where *I_P_* and *I_D_* are the photocurrent and dark current before avalanche multiplication, respectively, and *I_PM_* and *I_DM_* are the multiplied photocurrent and dark current after avalanche multiplication, respectively. In this work, the values of *I_P_* and *I_D_* are extracted under a 10 kV reverse bias, and the calculated results are shown in Figure 4. The results indicate that the avalanche gain values under various UV light intensities are nearly identical. In the avalanche multiplication region, the avalanche gain is primarily determined by the bias voltage. As the bias voltage increases, the avalanche gain increases rapidly. When the bias voltage approaches the breakdown voltage, the avalanche gain can exceed 10^5^. This high avalanche gain leads to significant photogenerated carrier multiplication and provides a higher photocurrent density, which is beneficial for improving the performance of the SiC LTT under weak UV illumination. To further investigate the impact-ionization-enhanced effect on the SiC LTT, both static and dynamic characteristics are simulated and analyzed.

## 3. Results and Discussion

**Figure 5:**
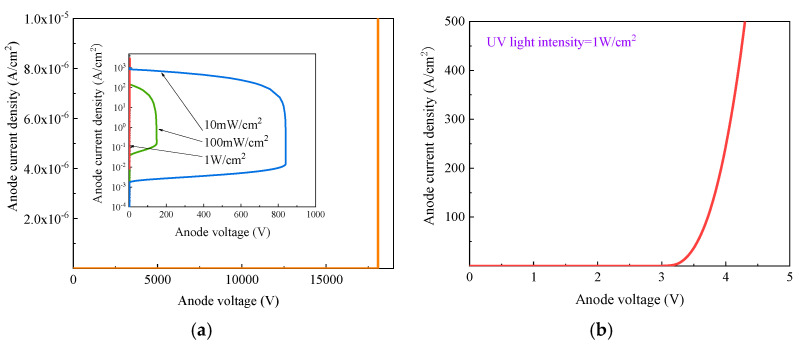
Static characteristic curves of the thyristor. (**a**) Blocking characteristics. (**b**) Turn-on characteristics.

The simulated static characteristics of the 4H-SiC LTT are shown in Figure 5. The forward blocking characteristic in Figure 5a indicates that the LTT can sustain a voltage up to 18.08 kV under dark conditions. The inset of Figure 5a shows the snapback behavior under UV illumination. The snapback voltage decreases with increasing UV light power intensity. As the UV intensity increases from 10 mW/cm^2^ to 100 mW/cm^2^, the snapback voltage drops from 840 V to 148 V. Under 1.0 W/cm^2^ UV irradiation, the LTT turns on directly. Figure 5b illustrates the forward conduction characteristics under a constant 1.0 W/cm^2^ UV illumination. The turn-on voltage of the LTT is approximately 3.2 V, and the on-state voltage drop at 100 A/cm^2^ is 3.72 V.

**Figure 6:**
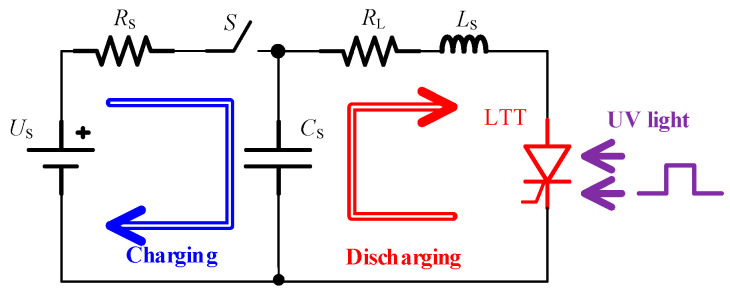
Diagram of the simulating circuit.

The dynamic characteristics of the 4H-SiC LTT are evaluated using a simulated load circuit, as illustrated in Figure 6. The test circuit consists of a charging loop and a discharging loop. A 100 nF capacitor C_S_ is used as the energy storage element, and a 100 Ω resistor R_S_ serves as the current-limiting component in the charging loop. In the discharging loop, a 100 nH inductor L_S_ and a 1.0 Ω resistor R_L_ are employed as load elements. The thyristor functions as the discharging switch in the circuit. During the test, the capacitor C_S_ is first charged to the preset bias voltage. Subsequently, the switch S is opened, and 365 nm UV light is applied to trigger the thyristor into conduction, initiating the discharge of the stored energy. Once the discharge current falls below the holding current, the thyristor returns to the blocking state.

**Figure 7:**
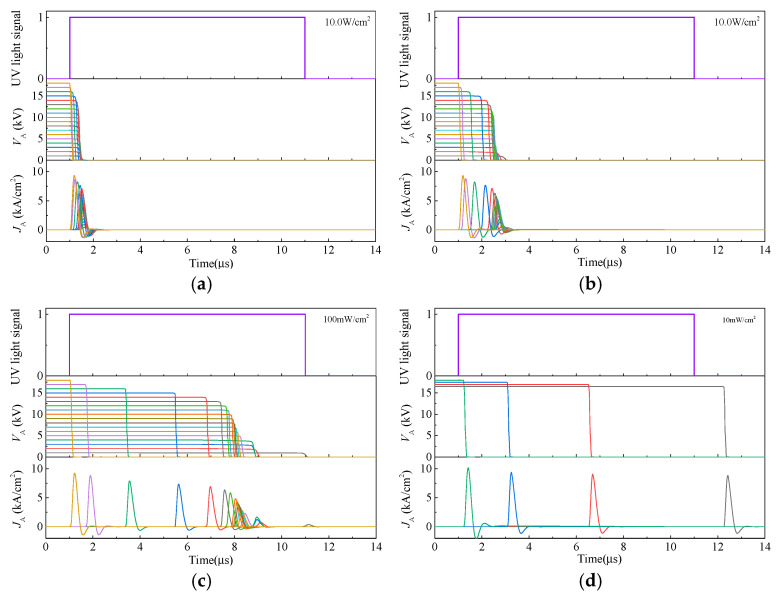
Dynamic waveforms of the 4H-SiC LTT under various bias voltages and UV light power intensities: (**a**)10.0 W/cm^2^; (**b**) 1.0 W/cm^2^; (**c**) 100 mW/cm^2^; and (**d**) 10 mW/cm^2^.

The simulated switching waveforms of the LTT are presented in Figure 7. The bias voltage V_A_, ranging from 1 kV to 18 kV, was applied between the anode and cathode electrodes through the storage capacitor C_S_, with a step increment of 1 kV. The UV light intensity was set to 10 W/cm^2^, 1.0 W/cm^2^, 100 mW/cm^2^, and 10 mW/cm^2^, respectively. The UV light duration was set to 10 μs. As shown in the figure, the SiC LTT is able to switch on at all bias voltage levels from 1 kV to 18 kV when the UV light power intensity is higher than 100 mW/cm^2^. When the UV light power intensity is lower than 10 mW/cm^2^, the SiC LTT can only switch on at 17 kV and higher bias voltages. Moreover, the smaller the power intensity of UV light, the greater the influence of the bias voltage on the switching-on characteristics. In particular, above 14 kV, the switching-on speed is enhanced rapidly with increasing bias voltage, which is applicable to all four light-triggered conditions. This is mainly caused by the impact-ionization-enhanced effect.

**Figure 8:**
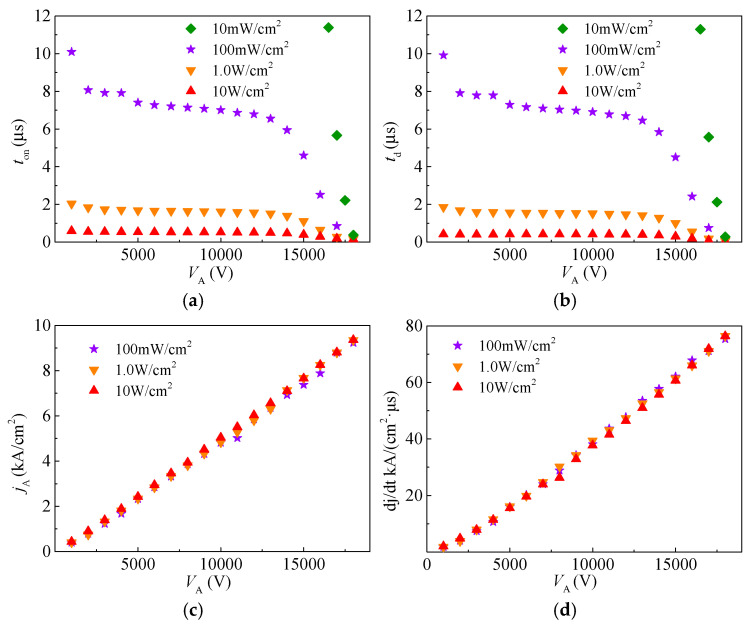
Dynamic characteristic parameters of the LTT at various bias voltages. (**a**) Turn-on time (*t*_on_). (**b**) Delay time (*t*_d_). (**c**) Peak current density (*j*_A_). (**d**) Current density rising rate (d*j*/d*t*).

To further elucidate the impact-ionization-enhanced effect on the switching performance of the SiC LTT, key characteristic parameters are extracted and shown in Figure 8. The turn-on time (*t*_on_) and delay time (*t*_d_) at different bias voltages and UV light power intensities are extracted and shown in Figure 8a,b. *t*_d_ is defined as the time from the initial UV light incident moment to 10% of the peak anode current density (*j*_A_) during the rising phase, while the *t*_on_ corresponds to the time at 90% of *j*_A_. The delay time is part of the turn-on time. As shown in the figures, both *t*_on_ and *t*_d_ decrease with increasing UV light power intensity. Furthermore, both *t*_on_ and *t*_d_ show the same trend of decreasing with increasing *V*_A_. This is because *t*_d_ accounts for a high proportion of the turn-on process in the SiC LTT, and *t*_on_ is mainly determined by *t*_d_. At 1 kV bias voltage, the *t*_d_ of the LTT under 100 mW/cm^2^, 1 W/cm^2^, and 10 W/cm^2^ are 9.9 μs, 1.8 μs, and 0.58 μs, respectively. At 18 kV bias voltage, the *t*_d_ of the LTT under 100 mW/cm^2^, 1 W/cm^2^, and 10 W/cm^2^ are 0.75 μs, 0.17 μs, and 0.05 μs, respectively. When the UV light intensity increases from 100 mW/cm^2^ to 10 W/cm^2^, *t*_d_ is reduced by about 9.32 μs at 1 kV bias voltage and 0.7 μs at 18 kV bias voltage, further confirming the substantial improvement in switching speed at higher bias voltages. Moreover, with the increase in bias voltage, both *t*_on_ and *t*_d_ first decrease, then remain stable, and finally decrease rapidly. This probably indicates that the mechanism of the SiC LTT’s turn-on process is different at different bias voltage ranges. *j*_A_ and the current density rising rate (d*j*/d*t*) at different bias voltages and UV light power intensities are extracted and shown in Figure 8c,d. As V_A_ increases, the peak current density *j*_A_ progressively increases, accompanied by a corresponding enhancement in d*j*/d*t*. Under a UV light intensity of 100 mW/cm^2^, when V_A_ is 1 kV, *j*_A_ reaches 399.2 A/cm^2^, corresponding to a d*j*/d*t* of 1.8 kA/μs. As V_A_ increases to 18 kV, *j*_A_ rises to 8787.7 A/cm^2^, with an associated d*j*/d*t* of 75.3 kA/μs. Both the *j*_A_ and d*j*/d*t* are primarily influenced by the bias voltage rather than the UV light intensity, where the *j*_A_ and d*j*/d*t* increase linearly with V_A_ increasing.

**Figure 9:**
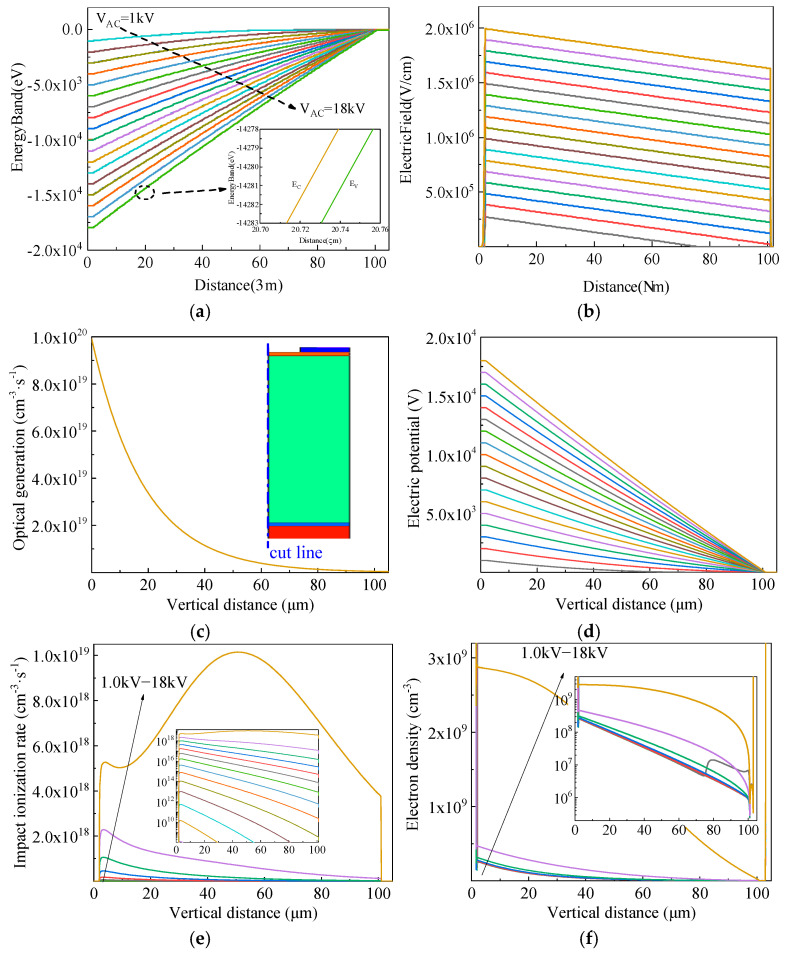
Internal parameters at the initial moment of the triggering process. (**a**) Energy band. (**b**) Electric field distribution. (**c**) Photogeneration distribution. (**d**) Potential distribution. (**e**) Impact ionization rate distribution. (**f**) Electron density distribution.

To further investigate the impact-ionization-enhanced effect in SiC LTTs and analyze the turn-on mechanism under varying bias conditions, key internal parameters at the initial stage of triggering under UV illumination of 100 mW/cm^2^ were simulated, as shown in Figure 9. The bias voltage ranged from 1 kV to 18 kV with a step of 1 kV. Figure 9a presents the energy band diagrams of the LTT at different bias levels, while Figure 9b shows the corresponding electric field distributions, indicating that the electric field is primarily concentrated in the space charge region of the J_2_ junction. Figure 9c illustrates the vertical distribution of the photogeneration rate under 100 mW/cm^2^ UV illumination. The photogeneration decreases exponentially from the surface of the thin n-base into the device interior. Figure 9d shows the vertical potential profiles at various bias voltages. Since the electric field is proportional to the slope of the potential, higher bias leads to stronger electric fields. Figure 9e depicts the vertical distribution of the impact ionization rate under different biases, consistent with the trend shown in Figure 3d. As expected, the ionization rate increases with the bias voltage, enhancing carrier multiplication during the UV-triggering process. Figure 9f shows the extracted electron concentration at the initial trigger moment. Although the number of photogenerated carriers is the same under all bias conditions, the electron concentration increases significantly with bias due to enhanced impact ionization, particularly at higher voltages. This leads to a higher initial current and a denser carrier plasma.

During device turn-on, photocarrier generation occurs mainly in the optical absorption region. As this region spatially overlaps with the high-field ionization zone, photogenerated carriers gain energy rapidly under the electric field and initiate secondary impact ionization, generating additional electron–hole pairs. Compared to dark-state ionization, this spatial overlap significantly enhances local carrier multiplication. Furthermore, even in partially overlapping regions, photogenerated carriers drift into the space charge region under the electric field and participate in ionization. In this manner, UV illumination not only increases the initial carrier density but also expands the effective ionization region. Overall, it enhances the positive feedback mechanism and accelerates the device turn-on process. Therefore, impact ionization enhancement provides a viable approach to achieve fast triggering of SiC LTTs under weak light conditions.

**Figure 10:**
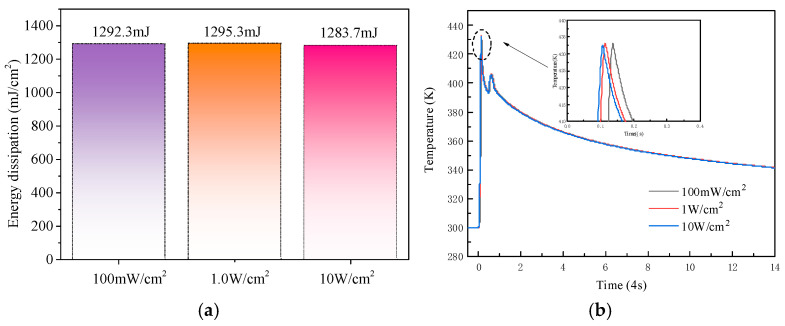
(**a**) Energy dissipation of the LTT under different UV light intensities. (**b**) Peak temperature of the device during turn-on.

In order to introduce the impact-ionization-enhanced effect into the turn-on process, the SiC LTT must operate under a relatively high bias voltage, close to the avalanche breakdown voltage. However, excessive energy dissipation during turn-on may lead to device failure. Therefore, controlling the energy dissipation during the turn-on process is critical for ensuring the safe operation of the SiC LTT. The energy dissipation during turn-on is calculated based on the transient current and voltage waveforms. At a bias voltage of 18 kV, the turn-on energy dissipations of the SiC LTT triggered by UV light intensities of 10 W/cm^2^, 1.0 W/cm^2^, and 100 mW/cm^2^ are shown in Figure 10a. The energy dissipation values under these three triggering conditions are similar, measured as 1283.7 mJ/cm^2^, 1295.3 mJ/cm^2^, and 1292.3 mJ/cm^2^, respectively. In addition, the device’s self-heating is considered when the bias voltage is 18 kV. The results are shown in Figure 10b. The device’s temperature remains around 433 K in all cases. Although an increase in temperature was observed, the device still operated within the safe thermal range without thermal runaway, showing stable and reliable performance under extreme conditions. The simulation results show that the operation at extremely high bias voltages does not cause the energy loss that SiC devices are unable to tolerate [25,26,27]. The SiC LTT is able to operate in an impact-ionization-enhanced mode.

## 4. Conclusions

The impact ionization effect is introduced into the SiC LTT to enhance photogenerated carrier multiplication during the switching-on process. The influence of impact ionization on the switching performance of the SiC LTT under weak UV light-triggering conditions is investigated through TCAD simulations. The results indicate that when the bias voltage exceeds 14 kV, the device enters the avalanche multiplication region, and the photocurrent significantly increases with the rising voltage under a fixed UV light intensity. Although the optical generation remains constant across different bias voltages, the initial electron density at high bias voltages is considerably higher than that at low bias voltages. The elevated electron density resulting from the impact ionization effect leads to a higher initial current density and shortens the turn-on time of the SiC LTT. Under a UV light intensity of 100 mW/cm^2^, the turn-on time of the thyristor decreases from 10.1 μs at 1 kV to 0.85 μs at 18 kV, while the corresponding current rise rate (d*j*/d*t*) increases from 1.8 kA/μs to 75.3 kA/μs. Moreover, the simulation results demonstrate that operation at extremely high bias voltages does not induce energy dissipation levels beyond what SiC devices can tolerate. Therefore, the impact-ionization-enhanced mechanism offers a promising approach for the rapid turn-on of SiC LTTs triggered by weak light.

## Data Availability

The data are contained within the article.

## Appendix A. Key Code Sections for TCAD Simulation

Math {

 Extrapolate

 Digits = 6

 ExtendedPrecision(128)

 ErrEff(electron) = 1e-32

 ErrEff(hole) = 1e-32

 RHSmax = 1e40

 RHSmin = 1e-35

 RHSfactor = 1e20

 Notdamped = 50

 Iterations = 45

 CdensityMin = 1e-30

}

Solve {

  Coupled(Iterations = 10,000 LineSearchDamping= 0.0001 { Poisson }

  Coupled(Iterations = 1000 LineSearchDamping= 0.001) { Poisson Hole }

  Coupled(Iterations = 1000 LineSearchDamping= 0.001) { Poisson Electron Hole }

  NewCurrentFile= “BV_”

   Quasistationary (

            Initialstep = 1e-6 Maxstep= 0.01 Minstep =1e-12

            Increment = 3.0 Decrement =5.0

      Goal { Name = “Anode” Voltage = 20,000 }

   ){ Coupled { Poisson Electron Hole } }

   }
